# Gut Microbiota and SCFAs Play Key Roles in QingFei Yin Recipe Anti-*Streptococcal* Pneumonia Effects

**DOI:** 10.3389/fcimb.2021.791466

**Published:** 2021-12-07

**Authors:** Xiaozhou Sun, Dandan Wang, Lina Wei, Lizhong Ding, Yinan Guo, Zhongtian Wang, Yibu Kong, Jingjing Yang, Liwei Sun, Liping Sun

**Affiliations:** ^1^ College of Chinese Medicine, Changchun University of Chinese Medicine, Changchun, China; ^2^ Research Center of Traditional Chinese Medicine, The Affiliated Hospital to Changchun University of Chinese Medicine, Changchun, China; ^3^ Center of Children’s Clinic, The Affiliated Hospital to Changchun University of Chinese Medicine, Changchun, China

**Keywords:** *Streptococcus pneumoniae*, pneumonia, QingFei Yin, gut microbiota, SCFAs, NF-κB-NLRP3

## Abstract

Emerging evidence has revealed the presence in animals of a bidirectional regulatory “lung-gut axis” that provides resistance to respiratory infections. Clues to the existence of this system stem from observations that respiratory infections are often accompanied by gastrointestinal symptoms, whereby intestinal microbiota appear to play pivotal roles in combating pathogenic infections. Importantly, short-chain fatty acids (SCFAs) produced by the gut microbiota appear to serve as the biological link between host immune defenses and gut flora. *Streptococcus pneumoniae* (*S.pn*), the main cause of lower respiratory tract infections, is involved in more than 1.189 million deaths per year. QingFei Yin (QFY) is known for its excellent therapeutic efficacy in combating bacterial lung infections. In this study, effects of *S.pn* infection on gut homeostasis were assessed using 16S RNA-based microbiota community profiling analysis. In addition, potential mechanisms underlying QFY recipe beneficial therapeutic effects against bacterial pneumonia were explored using *S.pn*-infected gut microbiota-depleted mice. Results of data analysis indicated that QFY treatment alleviated lung infection-associated pathogenic processes, while also promoting repair of disordered gut flora and counteracting *S.pn* infection-associated decreases in levels of SCFAs, particularly of acetate and butyrate. Mechanistically, QFY treatment suppressed inflammatory lung injury through inhibition of the host NF-κB-NLRP3 pathway. These results inspired us to identify precise QFY targets and mechanisms underlying QFY anti-inflammatory effects. In addition, we conducted an in-depth evaluation of QFY as a potential treatment for bacterial pneumonia.

## Highlights

• QFY treatment inhibited activation of the NF-κB-NLRP3 pathway by regulating gut microbial SCFAs that, in turn, facilitate recovery of mice from bacterial pneumonia. • In addition, this research confirmed the existence of a gut-lung axis and identified a potential mechanism underlying QFY immunomodulatory and pneumonia-alleviating therapeutic effects.

## Introduction

As the largest and most complex micro-ecosystem of the human body, the intestinal microbiota has emerged as a positive player in host defenses against pathogens, especially those involved in respiratory infections (McAleer and Kolls, 2018). In fact, the concept of crosstalk occurring between intestinal microbiota and the pulmonary immune system is currently widely accepted and is supported by recent observations. However, this concept is not new, but was documented 2000 years ago in the medical books of the *Yellow Emperor’s Classic of Internal Medicine*, where it was described as “The exterior-interior relationship between the lung and the large intestine.” What is very exciting is that modern science has uncovered molecular mechanisms in support of such a relationship, which is currently referred to as the “lung-gut axis” (Chakradhar, 2017; Wypych et al., 2019), with accumulating evidence supporting bidirectional communication between “lung” and “gut” (Sze et al., 2014). Indeed, many respiratory infections are accompanied by gastrointestinal symptoms. For instance, pulmonary infection has been shown to trigger disorder of gut bacteria that induce intestinal injury (Perrone et al., 2012). Vice versa, many gastrointestinal disorders are associated with respiratory tract manifestations, such as decreased lung function that often occurs in inflammatory bowel disease (IBD) patients during disease flareups (Douglas et al., 1989; Ceyhan et al., 2003). Moreover, respiratory symptoms can occur after intestinal injury, thus further supporting the claim that such symptoms may be a consequence of intestinal floral disruption.

Recently, it has been demonstrated that the intestinal microbiota plays a protective role in the host defense against pathogens, especially with regard to respiratory immunity. For example, mice with depleted gut microbiota showed increased bacterial dissemination and more severe injury, both of which were triggered by the host inflammatory response against *S.pn* infection (Schuijt et al., 2016). The results of this and other studies have prompted researchers to consider finding ways to induce or maintain gut microbiota homeostasis as a new potential therapeutic strategy for targeting pulmonary disease.

Short-chain fatty acids (SCFAs), including butyrate, propionate, and acetate, are derived from the metabolism of dietary fiber involving both the gut microbiota of the colon and the serum metabolome (Fukuda et al., 2011; Arpaia et al., 2013; Furusawa et al., 2013; Smith et al., 2013). SCFAs have been shown to exert pivotal protective effects on host immune homeostasis and defense (Lukasova et al., 2011), while a deficiency of dietary fiber may underlie development of asthma, allergies, and certain respiratory infectious diseases (Thorburn et al., 2015). In recent years, evidence in support of the importance of SCFAs and dietary fiber to health has accumulated, with results of some studies clarifying mechanisms underlying such effects. One such mechanism that is gaining support involves SCFAs-based inhibition of nuclear factor kappa-B (NF-κB) as a potential prerequisite pathway for preventing bacterial pneumonia (Vinolo et al., 2011).

*Streptococcus pneumoniae* (*S.pn*), a species of Gram-positive bacteria that colonizes the upper respiratory tract, is a major pathogen-induced cause of pneumonia, septicemia, and meningitis, particularly in young children (Quinton et al., 2018). Use of antibiotics has served as the major therapeutic strategy for combating bacterial infectious diseases, due to direct antimicrobial effects of these drugs (Quinton et al., 2018; Kadri and Boucher, 2020). However, their use is accompanied by gut flora disorder and emergence of drug-resistant bacteria (Bhalodi et al., 2019; Strati et al., 2021). Therefore, it is urgently important to discover other safe and effective therapeutic drugs to combat bacterial pneumonia. Traditional Chinese medicine (TCM) emphasizes holistic concepts and the balance between Yin and Yang associated with mutual relationships that have formed over millions of years. Indeed, numerous studies have demonstrated that ethnic medicines can reverse intestinal microecological disorders caused by pathogen infections (Wu and Tan, 2019; Lin et al., 2021). For instance, ethnomedicines such as berberine, baicalin, apigenin (Radulovic et al., 2018), and sulforaphene (Li et al., 2017) have been shown to restore normal gut flora balance after pathogen infection-induced disruption by increasing the abundance of probiotic organisms in the gut (Li et al., 2011; Wu et al., 2018; Zhang et al., 2021). One possible mechanism associated with beneficial effects of one of these medicines, baicalin, for protecting rats against gut microbiota imbalance was linked to modulation of SCFAs content (Zhu et al., 2020). Moreover, traditional Chinese medicine (TCM)-based polysacharides have been shown to exert immune-protectiveation by increasing abundances of certain beneficial gut bacteria, especially those belonging to phyla Bacteroidetes (genus *Prevotella*) and Firmicutes (genus *Ruminococcus*) and by enhancing production of SCFAs (Tang et al., 2018).

Qingfei Yin (QFY), a prescription used for the treatment of pneumonia, has been administered clinically for more than 60 years. QFY contains four Chinese herbs that *Scutellaria baicalensis* Georgi [Lamiaceae; Scutellariae radix], *Forsythia suspensa* (Thunb.) [Oleacea; Forsythiae Fructus], *Belamcanda chinensis* (L.) DC [Iridaceae; Elamcandae rhizoma], and *Fritillaria cirrhosa* D. Don [Liliaceae; Fritillaria Cirrhosae bulbus]. Our previous studies had revealed that QFY significantly alleviated pathological manifestations of lung infection and reduced cytokine levels in mice with *S.pn*-induced pneumonia, thus quenching inflammation-induced lung injury.

In this study, the important role of gut microbiota in infection, coupled with profound anti-inflammatory effects of SCFAs, inspired us to explore the gut-lung axis to discover targets of QFY treatment involved in alleviation of pneumonia. Here, we report that QFY treatment suppressed inflammatory injury by counteracting disorder of the gut microbiota induced by *S.pn* infection and associated decreased SCFAs levels, particularly of acetic acid and butyric acid. Mechanistically, this effect was dependent on inhibition of the NF-κB pathway by SCFAs. This research provides a rationale that supports future investigations to identify precise targets and mechanisms of QFY action toward the development of new medicines to treat lung infectious diseases.

## Materials and Methods

### Materials and Reagents

*S.pn* serotype 2 strain D39 was provided by Zunyi Medical College. QFY was provided by the Affiliated Hospital of Changchun University of Traditional Chinese Medicine (Jilin, China). Acetate (#695092) and butyrate (#B103500) were purchased from MilliporeSigma. Mouse interleukin-lβ (IL-1β, #MLB00C), interleukin-6 (IL-6, #M6000B), and tumor necrosis factor-alpha (TNF-α, #MTA00B) enzyme-linked immunosorbent assay (ELISA) kits were obtained commercially (R&D Systems, Minneapolis, MN, USA). Rabbit polyclonal antibodies against NF-κB P65 (#ab16502), NF-κB p-P65 (#ab194726), inhibitor of NF-κB (IκBα) (#ab7217), glyceraldehyde 3-phosphate dehydrogenase (GAPDH) (#ab8245) and β-tubulin (#ab18207) were purchased from Abcam (Cambridge, MA, USA). Rabbit polyclonal antibodies against NOD-like receptor protein 3 (NLRP3) (#15101S) and cleaved caspase-1 (cleaved-Casp1) (#89332S) were purchased from Cell Signaling Technology (Beverly, MA, USA).

### Preparation and Components Identification of QFY Aqueous Extract

QFY was provided by the Affiliated Hospital of Changchun University of Traditional Chinese Medicine (Jilin, China). Each component powder was accurately weighed and formulated to generate QFY based on the following ratio (by weight): *Scutellaria baicalensis* Georgi*, Forsythia suspensa* (Thunb.), *Belamcanda chinensis* (L.) DC, and *Fritillaria cirrhosa* D. Don of 8:8:8:3. According to the standard procedure (National Pharmacopoeia Committee, 2005), QFY powder was decocted 3 times with 200 mL of water at 100 °C to obtain the aqueous extract. All decocted solutions were combined and centrifuged then the supernatant was dried under vacuum to produce a brown powder. The extract was stored in the laboratory at −80°C until use. In previous studies, 19 main chemical compounds comprising QFY were detected using a method based on high performance liquid chromatography with tandem mass spectrometry (HPLC-MS/MS). Comparisons of results obtained from analysis of ten batches of QFY recipe indicated batch-to-batch consistency within the range of 95% to 98% of all major chemical constituents. Thus, different batches of QFY retained principal active components of the four Chinese herbal ingredients with acceptable batch-to-batch variability (**Figure S1**; **Tables S1, 2**).

### Bacterial Strains and Culture Conditions

The *S.pn* strain D39 (Berry et al., 1989) was cultured at 37°C in Todd-Hewitt broth then the culture was inoculated onto tryptic soy broth (TSB) agar plates and incubated under the same conditions overnight. Next, bacterial colonies were collected from the plates and cultured in Todd-Hewitt broth supplemented with 5% yeast extract until growth reached mid-log phase (as determined from the optical density of the culture at 600 nm).

### Animals

After female BALB/c mice (approved by the Animal Ethics Committee of Changchun University of Chinese Medicine-20190116) were received from the Experimental Animal Center of Changchun University of Traditional Chinese Medicine, they were housed and maintained for 6 weeks until they weighed 20 ± 2 g. Next, mice were allowed to rest for 5 d to acclimate before being subjected to experimental manipulations. For experimentation, mice were randomly assigned to nine groups: Control, Model, QFY (L) (0.21 g/kg), QFY (H) (0.42 g/kg), gut microbiota-depleted (DEPL), *S.pn*+DEPL, *S.pn*+DEPL+QFY, *S.pn*+DEPL+Acetate, and *S.pn*+DEPL+Butyrate (n = 7 per group). In experiments with DEPL mice, mice received high-dose QFY.

To deplete the gut microbiota, (designated as DEPL in group names above), mice were treated with a broad-spectrum antibiotic in drinking water as previously reported (Schuijt et al., 2016). Next, mice assigned to groups Model, QFY (L), QFY (H), *S.pn*+DEPL, *S.pn*+DEPL+QFY, *S.pn*+DEPL+Acetate, and *S.pn*+DEPL+Butyrate were lightly anesthetized *via* inhalation of isoflurane then were infected *via* left nasal inoculation of a 25-μL volume of nose drops containing 2.5 × 10^8^ CFU/mL of *S.pn* to establish the *in vivo* pneumonia model (Dessing et al., 2010). Beginning at the time of *S.pn* infection, microbiota-deficient mouse groups received drinking water containing a broad-spectrum antibiotic. Meanwhile, mice in QFY treatment groups QFY (L), QFY (H), and *S.pn*+DEPL+QFY were intragastrically administered QFY twice daily, while mice in the *S.pn*+DEPL+Acetate group were administered acetate (200 mM) and mice in the *S.pn*+DEPL+Butyrate group received butyrate (100 mM) in drinking water. All mice were sacrificed at 48 h post-infection.

### Mice Body Weight and Immune System Organ-Based Indices

Body weight, lung tissue wet weight to dry weight (W/D) ratio, and immune system-associated organ indices were determined in order to assess general characteristics of mice during experiments. Determinations of mouse organ immune index values (for thymus and spleen) were conducted using the following formulas: thymus index = (thymus weight/mouse weight) × 10, spleen index = (spleen weight/mouse weight) × 10.

### Micro-CT Scanning

As report previously (van Deel et al., 2016; Ruscitti et al., 2017), mice were anaesthetized with isoflurane then lung imaging was performed using a Quantum FX Micro-CT scanner (PerkinElmer, Inc., Waltham, MA). The X-ray system of the scanner uses a microfocus tube with a focal spot size of 5 μm at 4 W and produces X-rays delivered as a cone-shaped beam. Images were acquired using X-ray tube settings of 90 kVp and 160 μA. Projection radiographs were taken throughout the 360-degree gantry rotation cycle during a total scan time of 14 min.

### Histopathological Observations and Total Lung Inflammation Scores

Lung tissues of mice were prepared for histology and analyzed as described previously using the following steps: immersion of tissues in 4% paraformaldehyde followed by paraffin embedding, sectioning to generate slices, attachment of slices to glass slides, dewaxing of slices with xylene, immersion of slides in eosin staining solution, dewaxing of slices until transparent, and sealing of stained slices on slides. Total lung inflammation scores (TLISs) were determined as previously reported (Dessing et al., 2007). The entire lung surface was analyzed with respect to the following parameters: interstitial damage, vasculitis, peri-bronchitis, edema, thrombus formation, and pleuritis.

### ELISA

The trachea was exposed through a midline incision and bronchoalveolar lavage fluid (BALF) was harvested by instilling 1 mL of sterile isotonic saline into the lungs. BALF fluid was then tested for levels of IL-6, IL-1β, and TNF-α by ELISA.

### DNA Extraction and 16S rRNA Gene-Based High-Throughput Sequencing

Total microbial DNA was extracted using the OMEGA Soil DNA Kit (D5625-01) (Omega Bio-Tek, Norcross, GA, USA) from mouse cecal samples (n = 7). Next, PCR amplification of 16s rRNA gene V3-V4 was performed using forward primer 799F (5’-AACMGGATTAGATACCCKG-3’) and reverse primer 1193R (5’-ACGTCATCCCCACCTTCC-3’). PCR amplicons were quantified then were sequenced using the Illlumina MiSeq platform with MiSeq Reagent Kit v3 at Shanghai Personal Biotechnology Co., Ltd. (Shanghai, China). For microbiome community profiling evaluations, sequence data were analyzed using QIIME2 and R software packages (v3.2.0) (Bolyen et al., 2019).

### Western Blotting

30 µg of lung tissue lysate was loaded per lane then samples were electrophoretically separated using 10% SDS-PAGE gels. Primary antibodies specific for the following antigens were used at 1:1000 dilution: β-tubulin, GAPDH, NLRP3, cleaved-Casp1, NF-κB p65, p-NF-κB p65, and IκB. After blots were incubated with primary antibodies overnight at 4°C, they were washed then incubated with horseradish peroxidase-conjugated IgG secondary antibody (either goat anti-mouse or goat anti-rabbit) diluted 1:5000. Protein bands were visualized and analyzed using a chemiluminescent imaging system (FluorChem, ProteinSimple, San Jose, CA, USA).

### Statistical Analysis

All data are presented as means ± SD as calculated using Student’s t-test Comparisons between groups were conducted using one-way or two-way ANOVA followed by Bonferroni or Tukey’s post-tests as indicated. Differences were considered significant vs Control group, ^#^ *P* < 0.05, ^##^ *P* < 0.01; vs Model group, ^*^ *P* < 0.05, ^**^ *P* < 0.01; n.s is used to denote results that are not significant.

## Results

### Effects of QFY on General Characteristics of *S.pn*-Infected Mice

As shown in **Table 1**, as compared with the Control group, body weights and spleen and thymus indices were decreased in untreated infected mice by 48 h after *S.pn* infection. However, the lung W/D ratio was reduced after QFY treatment (*P* < 0.05), suggesting that QFY treatment of infected mice significantly reduced pulmonary edema and preserved host immune system balance during *S.pn* infection.

**Table 1:** Measurement of visceral index.

Group	Mice Weight (g)	Spleen index (mg/g)	Thymus index (mg/g)	Lung W/D Ratio
Control	19.30 ± 0.66	4.81 ± 0.45	3.15 ± 0.29	4.63 ± 0.63
Model	16.77 ± 1.72^#^	4.24 ± 0.64	2.27 ± 0.29^##^	7.74 ± 1.07^##^
QFY (L)	17.60 ± 1.41	4.11 ± 0.46	2.24 ± 0.19	6.85 ± 0.58
QFY (H)	17.89 ± 1.30	4.89 ± 0.94	2.82 ± 0.28^*^	5.30 ± 0.53^**^

Data were expressed as mean ± SD (n = 7). Significant difference from the Control group was designated as ^##^P < 0.01. Significant difference from the Model group was designated as *P < 0.05，**P < 0.01.

**Figure 1:**
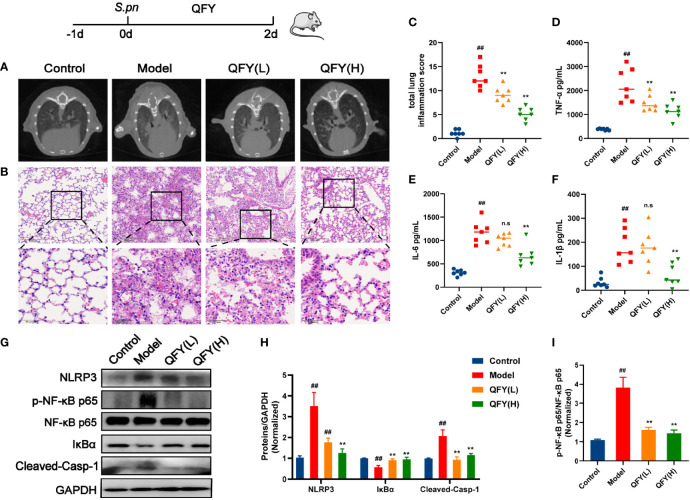
QFY prevented *S.pn*-induced lung inflammatory injury in infected mice. Model, QFY (L), and QFY (H) groups of mice were inoculated with 2.5 × 10^8^ CFU/mL *S.pn* in 25 μL for 48 h. **(A)** Micro-CT images are presented. **(B)** Pathologic and histopathological changes in lung tissues as revealed by H&E staining (original magnification 40×, scale bar: 20 μm). **(C)** Total lung inflammation score. **(D–F)** Detected levels of inflammatory cytokines IL-1β, IL-6, and TNF-α. **(G)** Western blot showing detected proteins. **(H, I)** Levels of quantified proteins on Western blots. Data are presented as the mean ± SD for each group. ^##^ *p* < 0.01 versus Control; **p < 0.01 versus Model; n.s, no significant differences.

Next, micro-CT scans and hematoxylin and eosin (H&E) staining were used to investigate lung inflammation induced by *S.pn* infection, as well as protective effects of QFY against lung tissue injury. In one micro-CT scan image, patchy, non-homogeneous high-density shadows and marginal haziness were observed in the lower lobe of the lung. Meanwhile, representative histological findings of tissues from intreated *S.pn*-infected mice showed extensive signs of pneumonia manifesting as deepened lung tissue color accompanied by signs of interstitial inflammation, vasculitis, bronchitis, and edema. In addition, lobes exhibited signs of congestion, swollen tissues, and evidence of neutrophil infiltration. Scans of lungs of infected animals receiving QFY treatment also exhibited patchy, non-homogeneous shadows, but these shadows were less dense than patches appearing in lung scan images of untreated infected animals. These imaging results indicated that lung tissues of QFY-treated infected mice exhibited less bleeding and swelling and more closely resembled lung tissues of healthy mice than did lung tissues of untreated infected mice (**Figures 1A, B**). Moreover, based on the TLISs semi-quantitative scoring system described in the Methods section, histopathological scores were much lower in the QFY-treated infected mouse group as compared with the untreated model group, indicating that QFY treatment of mice prevented inflammatory lung damage induced by *S.pn* infection (**Figure 1C**). Antibiotic treated also reduced the TLISs, but we observed that a considerable number of mice had diarrhea and loose feces which not observed in QFY group, regrettably (**Figure S2**).

In addition, we measured levels of inflammatory cytokines in mice BALF specimens. As shown in **Figures 1D–F**, *S.pn* infection led to significantly increased inflammatory cytokine levels of TNF-α, IL-1β, and IL-6 in BALF (*p* < 0.01), while QFY treatment of infected mice led to significantly decreased BALF levels of these cytokines. Thus, these results suggest that *S.pn* infection induced lung inflammation that was reversed by QFY treatment.

### Impact of QFY on Inflammation-Associated Proteins in the *S.pn*-Induced Mouse Pneumonia Model

Levels of NF-κB-NLRP3 pathway proteins that play key roles in lung immune responses were evaluated by Western blot analysis. As shown in **Figures 1G–I**, the NLRP3 signaling pathway was activated by *S.pn* invasion, as indicated by increased levels of NLRP3 and cleaved-Casp1 proteins. In addition, NF-κB, a transcription factor associated with NLRP3 pathway regulation, was also activated by *S.pn* infection, as confirmed by both an observed 43.32% decrease in IκBα level and a 3.79-fold increase in NF-κB p65 phosphorylation level as compared with corresponding Control group levels. These relative differences indicate that *S.pn* infection triggered a domino effect by activating an intense inflammatory cascade accompanied by induction of a mature cytokine response that induced high-level release of cytokines such as IL-1β. Strikingly, QFY treatment markedly maintained expression of these proteins at normal levels, especially in the QFY(H) group. Thus, hereafter data associated with QFY treatment groups was obtained using a high dose of QFY (0.42 g/kg) if not otherwise mentioned. Furthermore, after *S.pn* infection of mice, measurements of bacterial loads revealed no significant differences between QFY-treated and untreated groups (**Figure S3**).

Taken together, the abovementioned data revealed that *S.pn* infection-induced lung inflammation was reversed by QFY treatment and that this QFY therapeutic effect may be linked to inhibition of NLRP3 and NF-κB p65 pathways.

### QFY Altered Diversity and Composition of the Gut Microbiota

**Figure 2:**
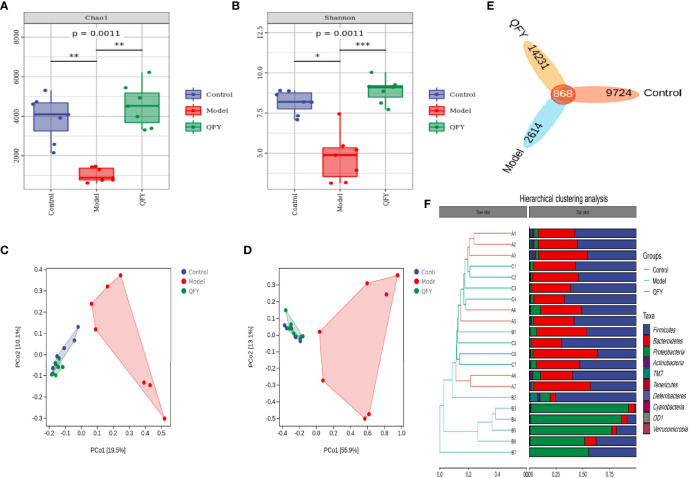
Alpha diversity metrics comparisons among Control, Model, and QFY groups using **(A)** Chao 1 indices, **(B)** Shannon indices, and **(C, D)** weighted and unweighted UniFrac distance-based PCoA, respectively. **(E, F)** The Venn diagram illustrating overlaps of phylum-level OTUs and the hierarchical cluster analysis results for each group. Data are presented as the mean ± SD for each group (*n* = 7), *p < 0.05, **p < 0.01 *versus* Control; **p < 0.01, ***p < 0.001 *versus* Model.

To explore the relationship between the inflammatory response and intestinal microecology, we analyzed the microbiota within mice cecal digesta. Community richness and diversity were evaluated using Chao 1 and Shannon indices (**Figures 2A, B**), with results showing that the Model group had markedly lower Shannon (*P* = 0.0011) and Chao 1 index (*P* = 0.0011) values as compared to corresponding Control group values. Importantly, QFY treatment restored infection-induced alterations of microbial community richness and diversity levels to nearly normal levels.

Principal coordinates analysis (PCoA) revealed overall differences in microbiota classification profiles among groups (**Figures 2C, D**), especially between Model and Control groups, while QFY treatment group results overlapped with those of the Control group. At the phylum level, the Venn diagram illustrating overlap of Operational taxonomic units (OTUs) profiling (**Figure 2E**) and hierarchical cluster analysis results (**Figure 2F**) revealed statistically significant differences in microbiota profiles between Control and Model groups, whereas the gut microbiota profile of the QFY-treated infected group more closely resembled that of the Control group. Taken together, the abovementioned results indicated that administration of QFY treatment shifted the microbiota profile from one of disease status towards one of healthy status.

**Figure 3:**
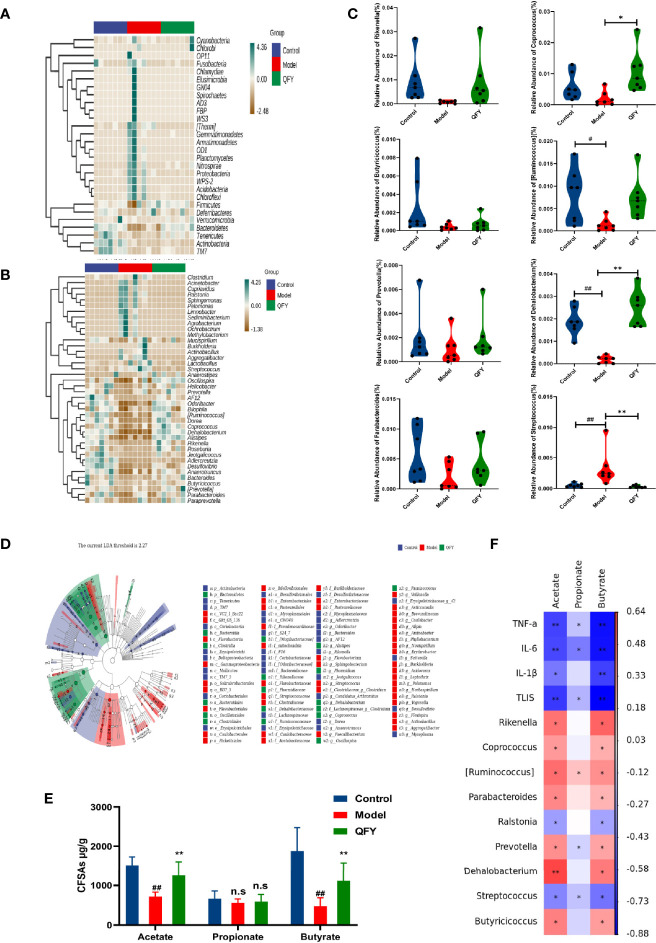
QFY Increased SCFAs and Shaped Mouse Gut Microbial Ecology. Mouse feces of Control, Model, and QFY groups were collected then analyzed to determine microbiota composition. **(A)** Relative fecal bacterial abundance percentages of the top 40 detected phylum. **(B)** Relative fecal bacterial abundance percentages of the top 40 detected geneus. **(C)** Box chart showing significant relative abundance variations at the genus level. **(D)** Classification tree of sample population based on LEfSe analysis, with sizes of nodes reflecting relative abundances and different colors corresponding to different taxonomic groups. **(E)** Levels of SCFAs (acetate, propionate, and butyrate) in mouse feces. **(F)** Spearman correlation analysis results for SCFAs and gut microbiota. Data are presented as the mean ± SD for each group (n = 7), ^#^ *p* < 0.05, ^##^ *p* < 0.01 *versus* Control; *p < 0.05, **p < 0.01 *versus* Model; n.s, no significant differences.

Next, in order to further compare species composition differences, heatmap were used to visualize significant bacterial taxonomic profile differences among groups at the phylum level (**Figure 3A**) and genus level (**Figure 3B**). In addition, LEfSe (LDA, linear discriminant effect size) was used to construct a hierarchical tree based on sample composition for use in difference analysis of results for all classification levels (**Figure 3D**).

*S.pn* infection caused significant perturbations of the microbiota at the phylum level (**Figures 2F**, **3A**). Phyla Firmicutes and Bacteroidetes dominated Control and QFY group profiles, while the phylum Proteobacteria was most prominent in the Model group profile. At the genus level, bacterial community profiles differed markedly between Control/QFY and Model groups (**Figure 3B**).

Notably, abundances of genera *Ralstonia*, *Sphingomonas*, *Ochrobactrum*, and *Streptococcus* were significantly increased after *S.pn* infection as compared to their respective abundances in Control and QFY groups (**Figure 3C**). Interestingly, SCFAs-producing bacterial genera mainly included *Coprococcus*, *Prevotella*, *Butyricicoccus*, [*Ruminococcus*], *Rikenella*, *Dehalobacterium*, and *Parabacteroides*, the levels of which were all decreased in untreated *S.pn*-infected mice and restored to normal levels in QFY-treated infected mice.

### QFY Treatment Reversed *S.pn* Infection-Induced Changes in Fecal SCFAs Contents

Observed differences in microbiota profiles among mouse groups in this study prompted us to test SCFAs levels in feces of each group (**Figure 3E**). As expected, *S.pn* infection decreased fecal levels of acetate and butyrate, while QFY treatment led to increases of both SCFAs to normal levels. Spearman correlation analysis revealed an interrelationship between SCFAs and corresponding gut microbiota among Control, *S.pn*-infected and QFY-treated *S.pn*-infected mice (**Figure 3F**). Intriguingly, highly significant negative correlations were observed between SCFAs and TLISs and between SCFAs and levels of cytokines IL-1β, IL-6, and TNF-α. Conversely, acetate and butyrate levels were each significantly positively correlated with abundances of genera *Coprococcus*, *Prevotella*, *Butyricicoccus*, [*Ruminococcus*], *Rikenella*, *Dehalobacterium*, and *Parabacteroides*, were significantly negatively correlated with abundances of *Ralstonia* and *Streptococcus*.

Taken together, alterations of gut microbiota composition and function may be attributed to effects of *S.pn* infection, which subsequently resulted in dysregulation of innate host immune responses. Conversely, QFY treatment led to marked reversal of infection-induced microbial imbalances to normalize the gut microbiota. This effect was positively correlated with increased levels of SCFAs acetate and butyrate and increased abundance of gut probiotic organisms that produce beneficial SCFAs.

### Gut Microbiota Depletion Exacerbated Lung Inflammation

**Figure 4:**
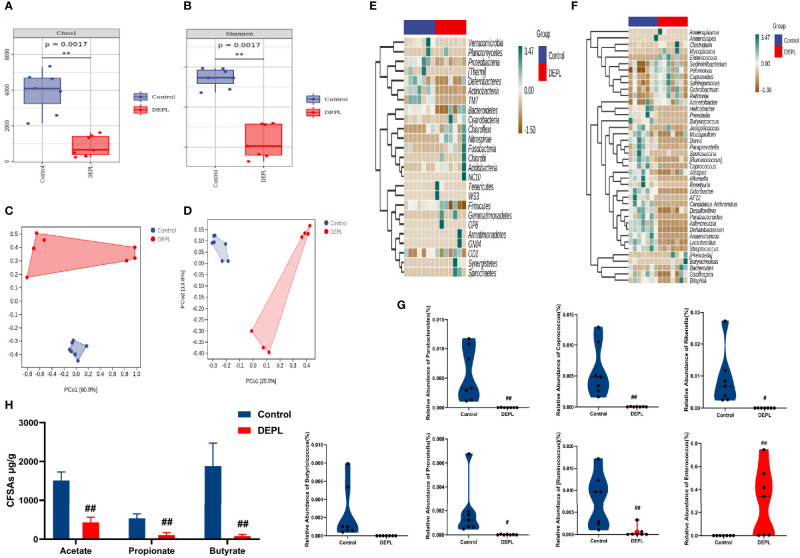
Alpha diversity metrics were compared between Control and gut microbiota-depleted mice using **(A)** Chao 1 indices **(B)** Shannon indices, and **(C, D)** weighted and unweighted UniFrac distance-based PCoA. **(E)** Relative fecal bacterial abundance percentages of top 40 phylum and **(F)** top 40 genus. **(G)** Box chart of genera showing significant relative variations in bacterial abundance percentages at the genus level. **(H)** Levels of SCFAs (acetate, propionate, and butyrate) in mouse feces. Data are presented as the mean ± SD for each group (n = 7), ^#^ *p* < 0.05, ^##^ *p* < 0.01, **p < 0.01 *versus* Control.

**Figure 5:**
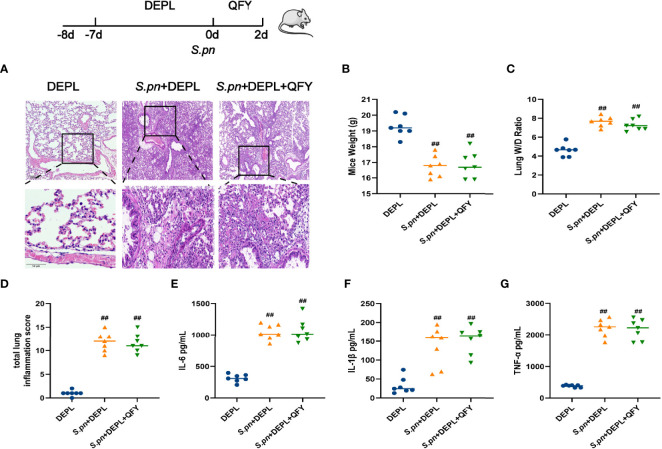
Mice assigned to DEPL, *S.pn*+DEPL, and *S.pn*+DEPL +QFY groups were first administered broad-spectrum antibiotics in drinking water then were intranasally inoculated with 2.5 × 10^8^ CFU/mL *S.pn* in a volume of 25 μL and monitored for 48 h. **(A)** Pathologic and histopathological changes in lung tissues as revealed by H&E staining (original magnification 40×, scale bar: 20 μm). **(B)** Total lung inflammation score. **(C)** Body weight and **(D)** lung W/D ratio of mice. **(E–G)** Levels of inflammatory cytokines IL-1β, IL-6, and TNF-α. Data are presented as the mean ± SD for each group, ^##^ *p* < 0.01 versus DEPL.

Mice were depleted by oral administration of broad-spectrum antibiotics in drinking water [as described previously (Schuijt et al., 2016)], we conducted all experiments as mentioned below in gut microbiota-depleted mice (termed DEPL mice). As shown in **Figure 4**, microbiota depletion was confirmed by observations of markedly lower gut microbial community richness and diversity in DEPL mice (**Figures 4A–D**). In addition, relative abundances of beneficial gut bacteria in DEPL mice were significantly decreased (**Figures 4E–G**), fecal SCFAs levels were decreased (**Figure 4H**), and DEPL mice exhibited more severe pneumonia symptoms (**Figure 5**). These results align with results reported recently showing that gut microbiota plays a protective role in the host defense against pneumococcal pneumonia (Schuijt et al., 2016). Taken together, the abovementioned results indicate that the gut microbiota-depleted mouse model was successfully established in this work. Moreover, QFY treatment of gut microbiota-depleted mice afflicted with *S.pn*-induced pneumonia exhibited normalization of microbiota profiles and associated metabolite levels, especially with regard to SCFAs. Thus, effects of SCFAs may play pivotal roles in observed QFY therapeutic effects associated with alleviation of pulmonary infectious diseases.

### Gut Microbiota Depletion Counteracted Beneficial Effects of QFY and SCFA Administration for Alleviating *S.pn*-Induced Lung Inflammation

**Figure 6:**
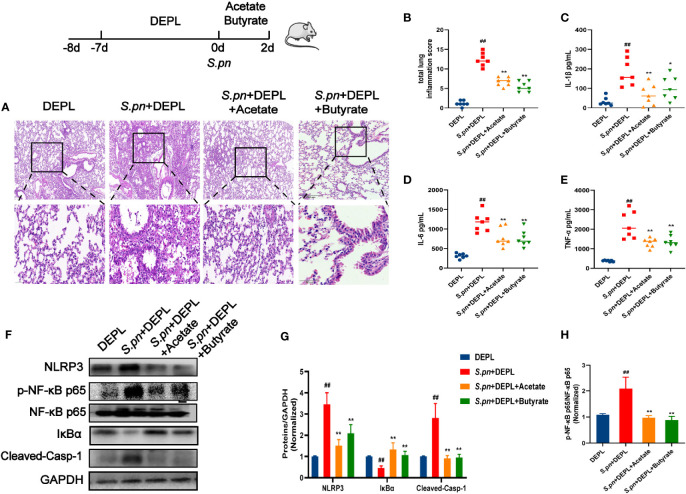
SCFAs Administration Alleviated Lung inflammation in DEPL Mice after *S.pn* Infection. DEPL, *S.pn*+DEPL, *S.pn*+DEPL +acetate, and *S.pn*+DEPL +butyrate groups of mice were administered broad-spectrum antibiotic in drinking water then were inoculated with 2.5 × 10^8^ CFU/mL *S.pn* 25 μL and monitored for 48 h. **(A)** Pathologic and histopathological changes in lung tissues as revealed by H&E staining (original magnification 40×, scale bar: 20 μm). **(B)** Total lung inflammation score. **(C–E)** Levels of inflammatory cytokines IL-1β, IL-6, and TNF-α in lung tissues. **(F)** Western blots revealing protein expression levels and **(G, H)** quantified protein levels determined from Western blot band areas and densities. Data are presented as the mean ± SD for each group, ^##^ *p* < 0.01 versus DEPL; *p < 0.05, **p < 0.01 *versus S.pn*+DEPL.

To investigate whether gut flora and SCFAs were potential targets of QFY pneumonia therapy, DEPL mice were treated with QFY after *S.pn* infection. **Figure 5** displays results demonstrating that QFY exhibited no therapeutic effect in mice with prior gut microbiota depletion induced by administration of a broad-spectrum antibiotic. Specifically, results of H&E staining and inflammatory cytokine level determinations revealed no differences between QFY-treated and untreated *S.pn*-infected DEPL mice, indicating that gut microbiota depletion counteracted the beneficial QFY effect. Based on the widely accepted fact that SCFAs fermented by gut flora are converted into metabolites with anti-inflammatory properties, we next administrated two SCFAs, acetate and butyrate, to *S.pn*-infected DEPL mice and observed marked changes associated with improved health status after SCFAs treatment. As shown in **Figure 6**, although QFY did not exert a protective effect in DEPL mice against pneumonia, a protective effect was achieved by administration of acetate and butyrate. More specifically, administration of these SCFAs improved the health of *S.pn*-infected DEPL mice as compared to untreated DEPL mice, as evidenced by decreased inflammatory cytokine levels and greater alleviation of pathology, as indicated by lung imaging findings.

It has been reported that SCFAs acting as NF-κB inhibitors block the inflammatory cascade (Chi et al., 2020; Wu et al., 2020). Therefore, we next evaluated the relative expression levels of proteins among experimental groups using Western blot analysis. As shown in **Figures 6F–H**, levels of NF-κB pathway proteins that were dramatically elevated after *S.pn* infection were decreased after acetate and butyrate treatments. However, in untreated infected mice, changes of levels of NLRP3, a target protein of NF-κB that plays a key role in immune responses to infectious diseases, paralleled changes of phosphorylated NF-κB levels, leading to cleavage of pro-caspase-1 and triggering of the inflammatory cascade.

In conclusion, we demonstrated that there is a close relationship between lung infection and disruption of intestinal flora. QFY therapeutic effects appeared to be associated with both repair of disordered intestinal flora through restoration of basal SCFAs levels to normal levels (in uninfected mice) and inhibition of NF-κB-NLRP3 pathway activity, which together alleviated lung injury induced by bacterial pneumonia.

## Discussion

Each year more than 0.65 million children younger than 5 years old and over 1.08 million adults die from lower respiratory infections (, 2018). Among all bacterial respiratory pathogens, *S. pneumoniae* (*S.pn*) is the most common cause of bacterial pneumonia (more than 35%), the main cause of lower respiratory tract infections, is involved in more than 1.189 million deaths per year (, 2018). Infections with this pathogen account for an extremely large proportion of overall worldwide disease burden and are the most frequent cause of pediatric hospitalizations (Rudan et al., 2008; Cilloniz et al., 2016; Oligbu et al., 2019). Pneumonia patient outcomes are not only dependent on inflammatory damage severity, but also on the intensity and nature of the host immune response (Quinton et al., 2018). Currently, clinical therapeutic strategies for treating pneumonia are unsatisfactory. Antimicrobials, previously considered fundamental interventional treatments for bacterial infectious diseases, are no longer viewed as completely beneficial treatments due to their harmful effects on the host microbiome and the emergence of antibiotic drug resistance due to drug misuse (Lee and Boucher, 2015; Bhalodi et al., 2019). Therefore, new safe and effective treatments for pneumonia are urgently needed.

Recently, multiple extra pulmonary organs have been shown to participate in host resistance to pulmonary infection, with special attention currently being paid to the role of the intestine in host immune responses to pathogens (Schuijt et al., 2013). Of note, mechanisms by which the intestine modulates host immunity and susceptibility to infection depend largely on gut microbiota (Rooks and Garrett, 2016).

Gut microbiota, the largest and most complex micro-ecosystem within the human body; maintains intestinal microecological equilibrium while regulating immune system function as part of a larger fine-tuned homeostatic system. Interestingly, the “lung-gut axis,” the existence of which is supported by recent epidemiological evidence, aligns with TCM theory of “The exterior-interior relationship between the lung and the large intestine.” Indeed, it is widely accepted that many respiratory infections are accompanied by gastrointestinal symptoms, with gut microbiota dysbiosis conferring increased susceptibility to respiratory disease (Litvak et al., 2018). For instance, gastrointestinal (GI) symptoms in COVID-19 patients are highly variable and can include diarrhea, bowel paralysis, constipation, and abdominal distension, among others, which are observed in 11% to 95% of patients (Biliński et al., 2021). Conversely, gut microbiota dysbiosis, which results from an unhealthy diet, pathogen infection, antibiotic usage, and other factors, appears to reduce systemic immune response capacity, further aggravating lung syndrome. IBS and IBD have higher susceptibility to pulmonary diseases (Douglas et al., 1989; Ceyhan et al., 2003; Wang et al., 2013).

Intriguingly, decreased gut microbiota diversity in infancy has been shown to increase risks of respiratory tract infection and asthma development (Abrahamsson et al., 2014). Moreover, it has been reported that gut microbiota plays a protective role in the host defense against *S.pn*-induced infection (Wu et al., 2020), with disordered gut microbiota previously shown to cause immune imbalances and aggravate lung inflammation (Schuijt et al., 2016). Therefore, gut flora has become a novel target of respiratory disease therapies currently under development. Toward this goal, herbal remedies and their active components, such as ginseng extract (Quan et al., 2020), plant polysaccharide (Chen et al., 2020), and baicalin (Wang et al., 2021), have been shown to exert prominent therapeutic effects that can improve immune defenses and maintain intestinal homeostasis.

Based on the abovementioned accumulated knowledge, we hypothesized that targeting of the gut microbiota and associated metabolites by herbal remedies may be used to alleviate bacterial pneumonia. Thus, here we evaluated the gut microbiota of mice using 16s rRNA sequencing of bacterial DNA isolated from mouse cecum contents. As expected, microbiota diversity decreased after *S.pn* infection, as evidenced by the decreased Chao and Shannon index values. Furthermore, gut microbiota composition dramatically changed after infection, as reflected by PCoA clustering and decreased relative abundances of fecal probiotic genera, including *Coprococcus*, *Prevotella*, *Butyricicoccus*, [*Ruminococcus*], *Rikenella*, *Dehalobacterium*, and *Parabacteroides* as compared with corresponding indicators for Control group mice. Strikingly, QFY treatment of *S.pn*-infected mice reversed infection-associated gut microbiota defects and restored microbiota bacterial profiles to a healthy state.

We also note here that QFY treatment of *S.pn*-infected mice not only regulated gut flora, but also alleviated general disease characteristics associated with *S.pn* infection, resulting in normalization of mouse body weight, lung W/D ratio, and thymus indices (**Table 1**). In addition, micro-CT and H&E staining showed decreased density of observed patchy non-homogeneous shadows, lung tissue bleeding, and lung tissue swelling after QFY treatment (**Figure 1**). More importantly, QFY reversed *S.pn* infection-induced lung inflammation and elevations of inflammatory cytokine levels in the lungs, with QFY effects found to be linked to inhibition of NLRP3 and NF-κB p65 pathways. Notably, up-regulation of expression of NLRP3, currently the most fully characterized inflammasome protein of the NLRs family, depends on activation of NF-κB pathway (Lemaitre et al., 1996a; Lemaitre et al., 1996b). Meanwhile, NF-κB is a transcription factor associated with expression of NLRP3 and cytokines that plays an important role in the inflammatory response after pathogen invasion (Schroder and Tschopp, 2010; Swanson et al., 2019). Specifically, after *S.pn* infection NLRP3 is activated then it binds to its adaptor protein ASC (apoptosis-associated speck-like protein containing a CARD) and caspase-1 (cysteinyl aspartate-specific proteinase-1), leading to production of inflammatory cytokines belonging mainly to the IL-1β-family (Swanson et al., 2019). Collectively, here QFY reduced NLRP3 expression by inhibiting activation of NF-κB, thus quenching the inflammatory response and lessening inflammation-induced injury, while also maintaining immune protection. Nevertheless, potential mechanisms underlying beneficial QFY effects and the relationship between the gut microbiota and the NF-κB-NLRP3 pathway remain unclear.

Recently, researchers have discovered that numerous metabolites produced by the host and the gut microbiota maintain the host immune system in a healthy homeostatic state (Dang and Marsland, 2019). Among these metabolites, SCFAs are the most important immunomodulatory metabolites present within the gut-lung axis and thus have attracted much attention from researchers in recent years (Tulic et al., 2016). Importantly, SCFAs have been shown to maintain and enhance intestinal mucosal epithelium integrity to counteract inflammation in intestinal and respiratory tracts by increasing the abundance and diversity of intestinal flora (Trompette et al., 2014). Furthermore, the ability of SCFAs to inhibit the NF-κB-NLRP3 pathway by preventing phosphorylation and degradation of IκBα is well known (Thorburn et al., 2014; Chi et al., 2020).

In this study, levels of SCFAs in mice cecal contents were determined to reveal the association between *S.pn*-infection and SCFAs levels. As expected, decreased levels of fecal SCFAs in *S.pn*-infected mice were accompanied by parallel decreased of gut probiotic abundance as well as exacerbation of pneumonia symptoms. Meanwhile, QFY treatment of infected mice led to increased fecal levels of acetate and butyrate by 1.74- and 2.32-fold as compared with corresponding levels for the Model group (**Figure 3E**). These changes were associated with increased abundance of probiotic genera that produce SCFAs, including *Coprococcus*, *Prevotella, Butyricicoccus, [Ruminococcus], Rikenella, Dehalobacterium*, and *Parabacteroides*. Based on the aforementioned results, we speculated that QFY may exert its protective effect against bacterial pneumonia-induced lung injury by targeting gut microbiota to stimulate production of SCFAs that inhibit the NF-κB/NLRP3 axis. To further confirm the NF-κB/NLRP3 axis as a QFY target involved in its mechanism of action, gut microbiota-depleted mice were generated here using a broad-spectrum antibiotic. This established method has been used previously to explore precise roles played by intestinal flora in human disease and to discover therapeutic targets (Gensollen et al., 2016). In this work DELP-mice exhibited aggravated lung injury and inadequate immune defenses after *S.pn* infection that agreed with previous research findings demonstrating a protective role of gut flora in alleviating bacterial pneumonia (Clarke et al., 2010). In recent years, emerging research has been focused on the association between complex herbal medicine formulations and microecology, with gut microbiota increasingly being recognized as a promising novel target of herbal medicines that improve the defensive capacity of the host. Our results here provide substantial evidence that gut microbiota and associated metabolites play pivotal roles in beneficial QFY treatment effects, as confirmed by the absence of a QFY therapeutic effect in DEPL mice (**Figure 6**). Notably, even after gut flora depletion, direct supplementation of mice with SCFA metabolites acetate or butyrate led to recovery from inflammatory injury induced by *S.pn* infection through inhibition of NF-κB-NLRP3 pathway activation to a certain extent.

Based on the abovementioned data, here we demonstrated for the first time that QFY treatment inhibited activation of the NF-κB-NLRP3 pathway by regulating gut microbial SCFAs that, in turn, facilitate recovery of mice from bacterial pneumonia. In addition, this research confirmed the existence of a gut-lung axis and identified a potential mechanism underlying QFY immunomodulatory and pneumonia-alleviating therapeutic effects.

## Data Availability Statement

The data presented in the study were deposited in the NCBI Sequence Read Archive repository, accession numbers SRP345072 & PRJNA778767.

## Ethics Statement

The animal experiment protocol (No. 20190116) was approved by the Changchun University of Chinese Medicine. Written informed consent was obtained from the owners for the participation of their animals in this study.

## Author Contributions

XS and DW performed the research, analyzed the data, and wrote the manuscript. LW, LD, and YG contributed to microbiome analysis. ZW, YK, and JY contributed to animal experiments. LWS revised the manuscript. LPS designed and funded the research, interpreted the data. All authors contributed to the article and approved the submitted version.

## Funding

This work was supported by the National Key Research and Development Program of China (No. 2017YFC1703202), Health and Health Technology Innovation Project of Jilin Province (No.2020J069), Jilin traditional Chinese medicine science and technology project (No.20211140), Natural Science Foundation of Jilin Province (No.20200201619JC), the National Natural Science Foundation of China (Nos. 82004099, No.82004421, No.81974579), the Jilin Scientific and Technological of Chinese Medicine Program (No. 2019023), the Department of Science and Technology of Jilin Province (NO. 20210101188JC), Inheritance and Innovation of Chinese Medicine of “Millions of Standouts” Project (the Project of Qihuang) of the Inheritance Workroom of the Chinese Medicine Master Wang Lie, the Jilin Scientific and Technological Program of Sanitation and Population Control (No. 2018J106), “Thirteenth Five-Year” Science and Technology Project of Jilin Provincial Education Department (No.JJKH20200889KJ), “Jujing cup” of Changchun University of traditional Chinese Medicine (No.YK202103) authorized support.

## Conflict of Interest

The authors declare that the research was conducted in the absence of any commercial or financial relationships that could be construed as a potential conflict of interest.

## Publisher’s Note

All claims expressed in this article are solely those of the authors and do not necessarily represent those of their affiliated organizations, or those of the publisher, the editors and the reviewers. Any product that may be evaluated in this article, or claim that may be made by its manufacturer, is not guaranteed or endorsed by the publisher.
