# Supplementary material for: Gut Microbiota and SCFAs Play Key Roles in QingFei Yin Recipe Anti-Streptococcal Pneumonia Effects
Source: Front Cell Infect Microbiol. 2021 Dec 7;11:791466. doi: 10.3389/fcimb.2021.791466 (PMC8688933; doi:10.3389/fcimb.2021.791466)
Supplement: Supplementary file 1 [file DataSheet_1.docx]

Supplementary Material

**1. Methods and material**

**1.1 Preparation of QFY and** [**component**](javascript:;)**s** [**identification**](javascript:;)

In order to identify compounds within the QFY extract, the extract was subjected to HPLC-MS/MS conditions using a chromatography system (Thermo, Ultimate 3000LC, Q Exactive HF), with separations conducted using a Zorbax Eclipse C18 column (1.8 μm, 2.1 × 100 mm). The mobile phase consisted of 0.1% formic acid water (phase A) and acetonitrile (phase B) with a flow rate of 0.3 mL/min, a 1:1 splitter ratio and gradient elution. Gradient change parameters for acetonitrile (B) were 0-2 min, 5% B; 2-6 min, 30% B; 6-7 min, 30% B; 7-12 min, 78% B; 12-14 min, 78% B; 14-17 min, 95% B; 17-20 min, 95% B; 20-21 min, 5% B; 21-25 min, 5% B. 2 μL of sample was injected at room temperature. The mass spectrometer was operated in both positive and negative ion modes. The following instrument parameters were applied: heater temperature of 325 °C, ion spray voltage of 3.5 kV, sheath gas flow velocity of 45 arb, auxiliary gas velocity of 15 arb, scavenging air velocity of 1 arb, and S-Lens RF Level of 55%. Scanning modes included full scan (*m/z* 100 ~ 1500) and data-dependent second-order mass spectrometry scanning (dd-MS2, TopN = 10). The collision mode was set to high energy collision dissociation (HCD). Compound Discoverer 3.1 was used for retention time correction, peak identification, peak extraction, etc. Compounds were identified based on comparisons to secondary mass spectrometry spectra of compounds within the thermo mzcloud online database and thermo mzvalut local database.

HPLC was conducted as described previously [[1-3](#_ENREF_36)]. The mixture was placed in a conical flask then it was mixed with 10 mL 70% ethanol. After placing a cork in the flask, the mixture was shocked for 40 min (power 250 W, frequency 50 kHz). After shocking, the mixture was centrifuged at 4000 rpm/min for 15 min to yield a supernatant containing QFY that was then filtered (0.22 μm). Ten batches of QFY compound from different sources were each separated using a ZORBAX Eclipse Plus C18 (4.6 mm × 250 mm, 5-µm) HPLC system (Shimadzu, Kyoto, Japan) and a diode array detector (UltiMate 3000, DIONEX, Sunnyvale, CA, USA). Analysis of specific components in each batch yielded chemical fingerprints (**Supplemental Table S1**). The mobile phase condition was recorded as follows: acetonitrile (as1122-801, Tedia, Fairfield, OH, USA) in water (A) and 0.05% phosphoric acid aqueous solution (B). The column temperature was 30 °C and the flow rate was 1.0 mL/min that allowed a gradient to form. UV detection was conducted at 266 nm. Empower software (Shimadzu) was used to collect and analyze chromatographic data.

**1.2 Measurement of Bacterial Loads**

The bacterial numbers were identified by colony counts of lung tissue smears as previously described [4; 5]. Lungs were collected from euthanized mice, and lung tissue homogenates were prepared in 1 ml of sterile PBS at 4°C and used to calculate the bacterial colony counts through the serial dilution method and smearing on solid media.

**2. Results**

**2.1 HPLC chromatograms of QFY formulations**

HPLC-MS/MS methods have been widely used to analyze major compounds in various herbal medicines. In this study, 19 main compounds of QFY were identified simultaneously. The total ion current (TIC) of these 19 components were shown in **Figures S1A, B**. for the exact identification of QFY, compound discoverer 3.1 was used for retention time correction, peak identification and extraction. Information about the compound’s name, molecular formula, retention time (RT), mass accuracy and adduct were listed in **Supplemental Table S2**. Consistent with HPLC-MS/MS result, 14 main peaks in the HPLC fingerprint were attributed to QFY compounds (**Figure S1C**)**.** Due to the fact that the proportion of the Cytidine, Adenosine, Caffeic acid, Phillyrin, monopalmitin were very low, the peaks of them were not identified in the HPLC, and they were not used as a reference for similarity evaluations. Finally, ten batches of QFY recipe were compared and analyzed. All the compounds similarities were within the range of 95% to 98%. Above results indicated that QFY retained the principal active component of four Chinese herbs, and formulations exhibited good repeatability (**Figure S1D**).


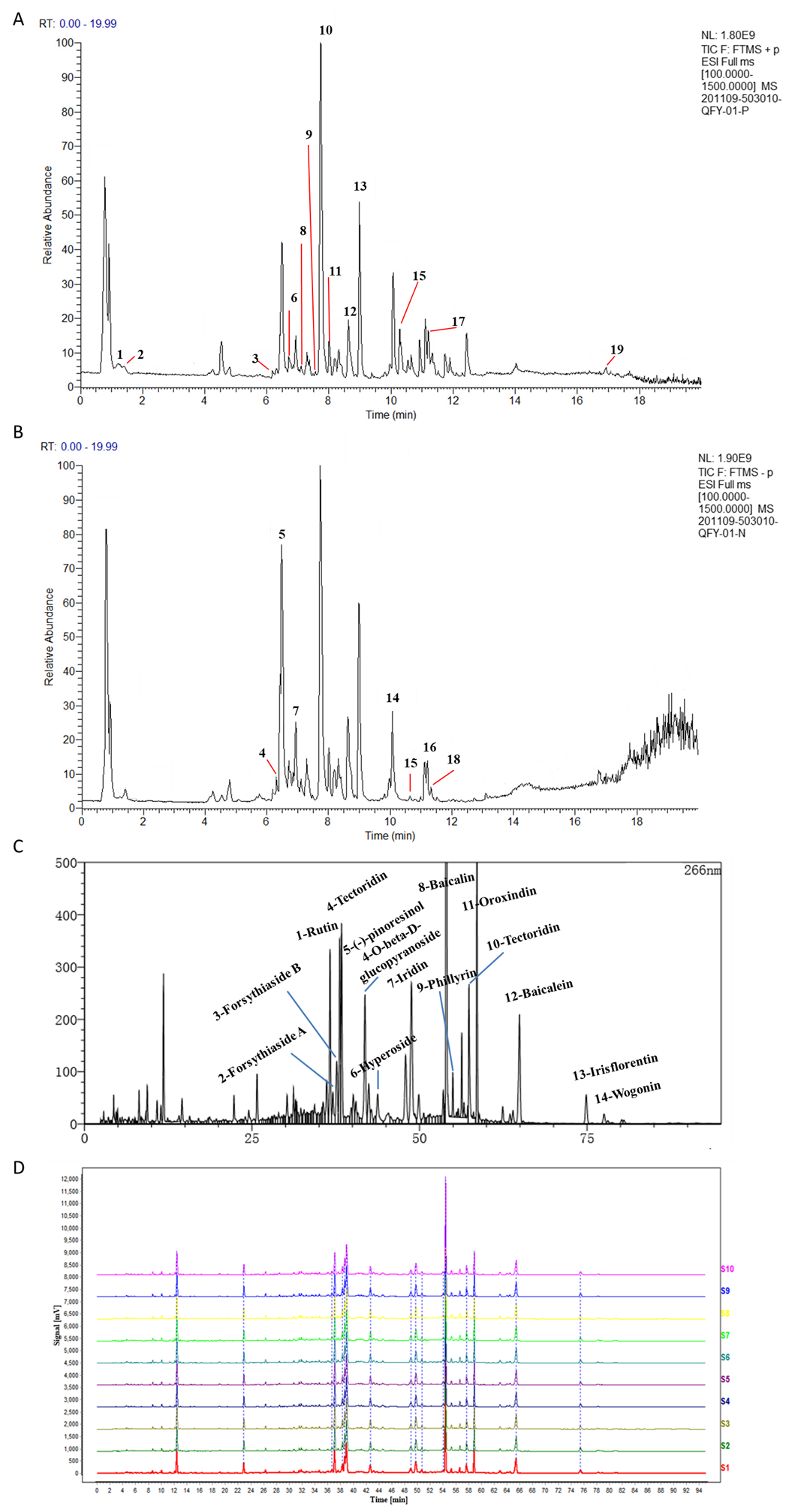


**Figure S1. HPLC chromatograms of QFY formulations.** Total Ion Current (TIC) of QFY formulation extracts in both positive **(A)** and negative **(B)** ion models. The peaks of 1–19 are listed in Table 1. **(C)** HPLC chromatogram of QFY formulation. **(D)** HPLC fingerprint chromatograms of 10 batches of QFY (S1–10) formulations analyzed using National Pharmacopeia Committee Chinese Medicine Fingerprint Similarity Evaluation System (2004A) software, with UV detection at 266 nm.

**Table S1 Source of 10 batches of QFY**

| **batch** | **medicinal materials** | **batch number** | **Manufacturer** |
| --- | --- | --- | --- |
| **S1** | **Belamcandae Rhizoma** | **A190019220** | **Peili (Nanning) Pharmaceutical Co., Ltd** |
|  | **Frsythia** | **A1701862** | **Peili (Nanning) Pharmaceutical Co., Ltd** |
|  | **Fritillaria Cirrhosa** | **A1801140** | **Peili (Nanning) Pharmaceutical Co., Ltd** |
|  | **Scutellaria baicalensis** | **A190116910** | **Peili (Nanning) Pharmaceutical Co., Ltd** |
| **S2** | **Belamcandae Rhizoma** | **18026374** | **Jiangyin Tianjiang Pharmaceutical Co., Ltd** |
|  | **Forsythia** | **19046344** | **Jiangyin Tianjiang Pharmaceutical Co., Ltd** |
|  | **Fritillaria Crrhosa** | **19056464** | **Jiangyin Tianjiang Pharmaceutical Co., Ltd** |
|  | **Scutellaria baicalensis** | **19046494** | **Jiangyin Tianjiang Pharmaceutical Co., Ltd** |
| **S3** | **Belamcandae Rhizoma** | **0065825524** | **Sichuan new green Pharmaceutical Technology Development Co., Ltd** |
|  | **Forsythia** | **0065825482** | **Sichuan new green Pharmaceutical Technology Development Co., Ltd** |
|  | **Fritillaria Cirrhosa** | **1812033** | **Sichuan new green Pharmaceutical Technology Development Co., Ltd** |
|  | **Scutellaria baicalensis** | **0065825521** | **Sichuan new green Pharmaceutical Technology Development Co., Ltd** |
| **S4** | **Belamcandae Rhizoma** | **A1800070** | **Nongbenfang 1** |
|  | **Forsythia** | **A1701863** | **Nongbenfang 1** |
|  | **Fritillaria Cirrhosa** | **A1801141** | **Nongbenfang 1** |
|  | **Scutellaria baicalensis** | **A190116911** | **Nongbenfang 1** |
| **S5** | **Belamcandae Rhizoma** | **19036074** | **Jiangyin Tianjiang Pharmaceutical Co., Ltd** |
|  | **Forsythia** | **19046344** | **Jiangyin Tianjiang Pharmaceutical Co., Ltd** |
|  | **Fritillaria Cirrhosa** | **19056464** | **Jiangyin Tianjiang Pharmaceutical Co., Ltd** |
|  | **Scutellaria baicalensis** | **19046494** | **Jiangyin Tianjiang Pharmaceutical Co., Ltd** |
| **S6** | **Belamcandae Rhizoma** | **0102942438** | **Sichuan new green Pharmaceutical Technology Development Co., Ltd** |
|  | **Forsythia** | **0102942435** | **Sichuan new green Pharmaceutical Technology Development Co., Ltd** |
|  | **Fritillaria Cirrhosa** | **1812033** | **Sichuan new green Pharmaceutical Technology Development Co., Ltd** |
|  | **Scutellaria baicalensis** | **0102942426** | **Sichuan new green Pharmaceutical Technology Development Co., Ltd** |
| **S7** | **Belamcandae Rhizoma** | **A1800069** | **Nongbenfang 2** |
|  | **Forsythia** | **A1701862** | **Nongbenfang 2** |
|  | **Fritillaria Cirrhosa** | **A1801140** | **Nongbenfang 2** |
|  | **Scutellaria baicalensis** | **A190116910** | **Nongbenfang 2** |
| **S8** | **Belamcandae Rhizoma** | **A190053610** | **Nongbenfang 3** |
|  | **Forsythia** | **A190053610** | **Nongbenfang 3** |
|  | **Fritillaria Cirrhosa** | **A190014310** | **Nongbenfang 3** |
|  | **Scutellaria baicalensis** | **A190053610** | **Nongbenfang 3** |
| **S9** | **Belamcandae Rhizoma** | **A190019220** | **Peili (Nanning) Pharmaceutical Co., Ltd** |
|  | **Forsythia** | **A1701832** | **Peili (Nanning) Pharmaceutical Co., Ltd** |
|  | **Fritillaria Cirrhosa** | **A1801120** | **Peili (Nanning) Pharmaceutical Co., Ltd** |
|  | **Scutellaria baicalensis** | **A190114210** | **Peili (Nanning) Pharmaceutical Co., Ltd** |
| **S10** | **Belamcandae Rhizoma** | **18074376** | **Jiangyin Tianjiang Pharmaceutical Co., Ltd** |
|  | **Forsythia** | **19046368** | **Jiangyin Tianjiang Pharmaceutical Co., Ltd** |
|  | **Fritillaria Cirrhosa** | **19056467** | **Jiangyin Tianjiang Pharmaceutical Co., Ltd** |
|  | **Scutellaria baicalensis** | **19046494** | **Jiangyin Tianjiang Pharmaceutical Co., Ltd** |

**Table S2. In the compound discover 3.1 library for the analysis of 19 compounds using UPLC-MS/MS**

| **No** | **RT**  **(min)** | **Identification** | **Molecular Formula** | **Expected Neutral Mass(Da)** | **Observed Neutral Mass(Da)** | **LC/MS**  **(ESI-)**  **(m/z)** | **Mass Accuracy (ppm)** | | **Adducts** |
| --- | --- | --- | --- | --- | --- | --- | --- | --- | --- |
| **1** | **1.189** | **Cytidine** | **C_9_ H_13_ N_3_ O_5_** | **243.08544** | **243.08552** | **244.0926** | **-0.33** | **[M+H]^+^** | |
| **2** | **1.588** | **Adenosine** | **C_10_ H_13_ N_5_ O_4_** | **267.09645** | **267.09675** | **268.10279** | **-1.12** | **[M+H]^+^** | |
| **3** | **6.113** | **Caffeic acid** | **C_9_ H_8_ O_4_** | **180.04215** | **180.04226** | **181.04947** | **-0.61** | **[M+H]^+^** | |
| **3** | **5.792** | **Caffeic acid** | **C_9_ H_8_ O_4_** | **180.04214** | **180.04226** | **179.03421** | -**0.62** | **[M-H]^-^** | |
| **4** | **6.345** | **Forsythoside B** | **C_34_ H_44_ O_19_** | **756.24826** | **756.24768** | **755.24115** | **0.77** | **[M-H]^-^** | |
| **5** | **6.522** | **Rutin** | **C_27_ H_30_ O_16_** | **610.15381** | **610.15338** | **609.14642** | **0.70** | **[M-H]^-^** | |
| **6** | **6.82** | **Forsythoside A** | **C_29_ H_36_ O_15_** | **624.20518** | **624.20542** | **625.21252** | **-0.38** | **[M+H]^+^** | |
| **7** | **6.983** | **(-)-pinoresinol 4-O-beta-D-glucopyranoside** | **C_26_ H_32_ O_11_** | **520.19442** | **520.19446** | **519.18713** | **-0.08** | **[M-H]^-^** | |
| **8** | **7.058** | **Hyperoside** | **C_21_ H_20_ O_12_** | **464.09513** | **464.09548** | **465.10214** | **-0.75** | **[M+H]^+^** | |
| **9** | **7.661** | **Iridin** | **C_24_ H_26_ O_13_** | **522.13725** | **522.13734** | **523.14453** | **-0.17** | **[M+H]^+^** | |
| **10** | **7.723** | **Phillyrin** | **C_27_ H_34_ O_11_** | **534.2086** | **534.210112** | **535.19586** | **-2.83** | **[M+H]^+^** | |
| **11** | **8.093** | **Baicalin** | **C_21_ H_18_ O_11_** | **446.08431** | **446.08491** | **447.09115** | **-1.34** | **[M+H]^+^** | |
| **12** | **8.729** | **Tectoridin** | **C_22_ H_22_ O_11_** | **462.11591** | **462.116212** | **463.12323** | **-0.65** | **[M+H]^+^** | |
| **13** | **8.986** | **Oroxindin** | **C_22_ H_20_ O_11_** | **460.10008** | **460.10056** | **461.10733** | **-1.04** | **[M+H]^+^** | |
| **14** | **10.118** | **Baicalein** | **C_15_ H_10_ O_5_** | **270.0528** | **270.05282** | **269.04553** | **-0.07** | **[M-H]^-^** | |
| **15** | **10.03** | **Tectorigenin** | **C_16_ H_12_ O_6_** | **300.06326** | **300.06339** | **299.05588** | **-0.43** | **[M-H]^-^** | |
| **15** | **10.341** | **Tectorigenin** | **C_16_ H_12_ O_6_** | **300.06308** | **300.06339** | **301.01037** | **-1.03** | **[M+H]^+^** | |
| **16** | **11.197** | **Chrysin** | **C_15_ H_10_ O_4_** | **254.05766** | **254.05791** | **253.05038** | **-0.98** | **[M-H]^-^** | |
| **17** | **11.283** | **Irisflorentin** | **C_20_ H_18_ O_8_** | **386.09958** | **386.100168** | **387.10693** | **-1.52** | **[M+H]^+^** | |
| **18** | **11.384** | **Wogonin** | **C_16_ H_12_ O_5_** | **284.06839** | **284.06847** | **283.0611** | **-0.28** | **[M-H]^-^** | |
| **19** | **17.467** | **monopalmitin** | **C_19_ H_38_ O_4_** | **330.27645** | **330.27701** | **331.28357** | **-1.69** | **[M+H]^+^** | |

**2.2 Effects of antibiotic treatment on *S.pn*-Infected Mice**

H&E results indicated that lung tissues of cefatriaxone (CTX) -treated infected mice exhibited less bleeding and swelling and more closely resembled lung tissues of healthy mice than did lung tissues of untreated infected mice (**Figure S2A**). Moreover, based on the TLISs semi-quantitative scoring system described in the Methods section, histopathological scores were much lower in the CTX-treated infected mouse group as compared with the untreated model group, indicating that QFY treatment of mice prevented inflammatory lung damage induced by *S.pn* infection and reduced the TLISs (**Figure. S2B**). However, we observed that a considerable number of mice had diarrhea and loose feces which not observed in QFY group.


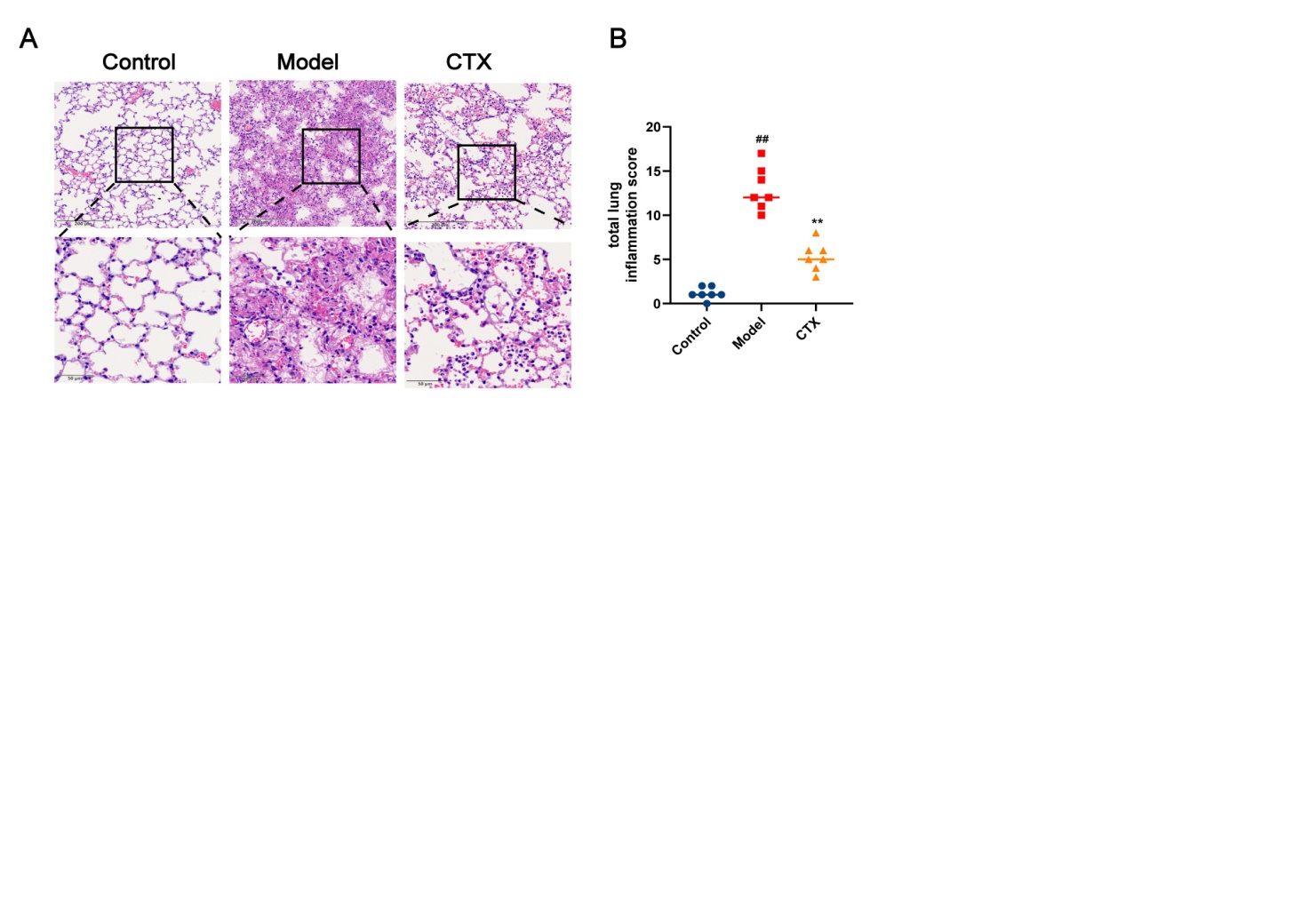


**Figure S2. CTX prevented *S.pn*-induced lung inflammatory injury in infected mice.** Model and CTX groups of mice were inoculated with 2.5 × 10^8^ CFU/mL *S.pn* in 25 μL for 48 h. **(A)** Pathologic and histopathological changes in lung tissues as revealed by H&E staining (original magnification 40×, scale bar: 20 μm)**. (B)** Total lung inflammation score. Data are presented as means ± SD, ^##^*p* < 0.01 versus Control, ^**^*p*< 0.01 versus Model.

2.3 **The effect of QFY on bacteria loading in *S.pn* infected mice.**

To evaluate the *S. pneumoniae* survival influenced by QFY, we determined the colony-forming units (CFU) and DNA content after administration of *S.pn* after 48 hours**.** The bacterial loading in the lungs was slightly lower in the QFY-treat high dose group than that of *S.pn* group with no significant differences (**Figures S3A, B**). Hence, we believed that inhibition of the bacteria survival is not the main pathway on which QFY exerts its therapeutic effect.

**
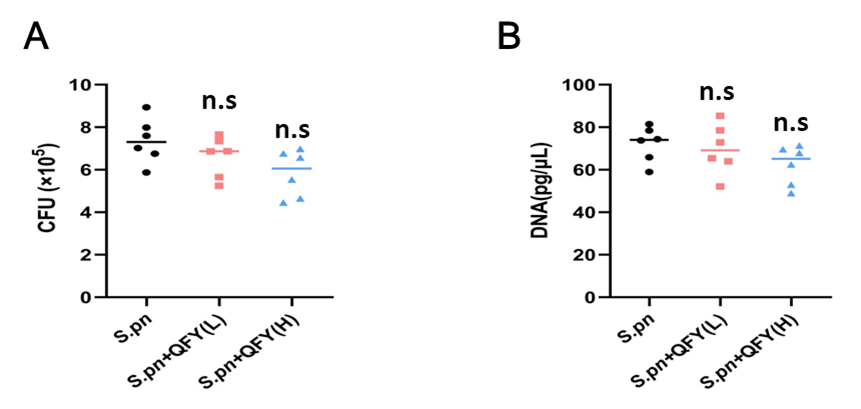
**

**Figure S3** The influence of QFY exerted on the lung bacteria burden of infected mice. **(A, B)** The infected extent could be estimated by CFU and DNA of the lung bacteria of the mice that had been influenced for 48 hours. Data are presented as means ± SD, n.s, no significant differences.

[**References**](javascript:;)

[1] D. Wang, H.K. Wong, L. Zhang, G.M. Mcalonan, X.M. Wang, S.C.W. Sze, Y.B. Feng, Z.J. Zhang, Not only dopamine D2 receptors involved in Peony-Glycyrrhiza Decoction, an herbal preparation against antipsychotic-associated hyperprolactinemia, Progress in Neuropsychopharmacology & Biological Psychiatry 39(2) (2012).

[2] Q. Huang, T. Lan, J. Lu, H. Zhang, D. Zhang, T. Lou, P. Xu, J. Ren, D. Zhao, L. Sun, DiDang Tang Inhibits Endoplasmic Reticulum Stress-Mediated Apoptosis Induced by Oxygen Glucose Deprivation and Intracerebral Hemorrhage Through Blockade of the GRP78-IRE1/PERK Pathways, Frontiers in Pharmacology 9 (2018).

[3] T. Li, S. Zhuang, Y. Wang, Y. Wang, W. Wang, H. Zhang, L. Chen, D. Wang, Z. Zhou, W. Yang, Flavonoid profiling of a traditional Chinese medicine formula of Huangqin Tang using high performance liquid chromatography, Acta Pharmaceutica Sinica B (2016) 148-157.

[4] X. Zhao, H. Li, J. Wang, Y. Guo, B. Liu, X. Deng, and X. Niu, Verbascoside Alleviates Pneumococcal Pneumonia by Reducing Pneumolysin Oligomers. Molecular pharmacology 89 (2016) 376-87.

[5] T. Isono, and H. Domon, Treatment of severe pneumonia by hinokitiol in a murine antimicrobial-resistant pneumococcal pneumonia model. 15 (2020) e0240329.

**
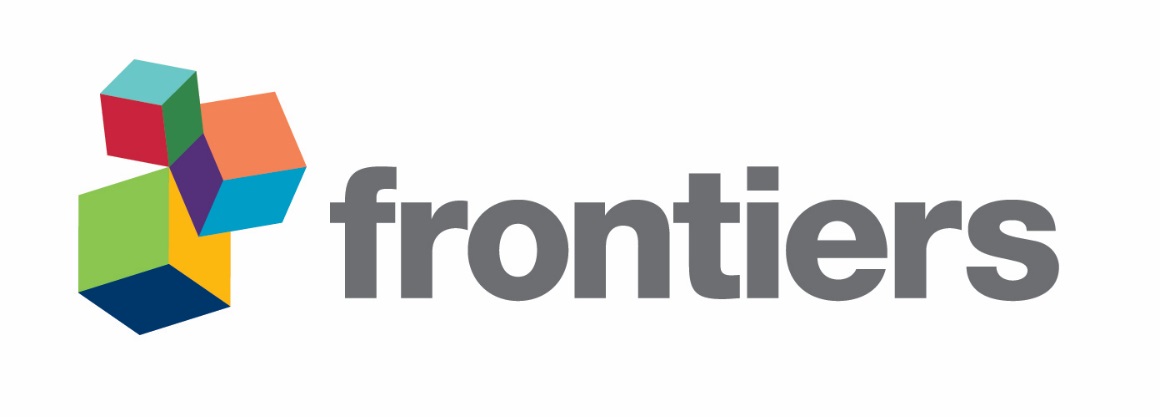
**
